# Development and validation of a novel immune-related prognostic signature in lung squamous cell carcinoma patients

**DOI:** 10.1038/s41598-022-23140-w

**Published:** 2022-12-01

**Authors:** Xianyu Liu, Deze Zhao, Yunhan Shan, Weifang Cui, Qun Xie, Junjie Jiang, Wei Peng, Chunfang Zhang, Chaojun Duan

**Affiliations:** 1 Department of Thoracic Surgery, Xiangya Hospital, Central South University, Changsha, Hunan China; 2 Hunan Engineering Research Center for Pulmonary Nodules Precise Diagnosis & Treatment, Changsha, Hunan China; 3 Department of Ultrasonic Imagine, Affiliated Hospital of Hunan Traditional Chinese Medicine Research, Changsha, Hunan China; 4 Department of Oncology, Hunan Provincial People’s Hospital, the First Affiliated Hospital of Hunan Normal University, Changsha, Hunan China; 5 National Clinical Research Center for Geriatric Disorders, Changsha, Hunan China; 6 Institute of Medical Sciences, Xiangya Lung Cancer Center, Xiangya Hospital, Central South University, Xiangya Road 87Th, Changsha, 410008 Hunan China

**Keywords:** Lung cancer, Tumour biomarkers, Tumour immunology

## Abstract

Lung Squamous Cell Carcinoma (LUSC) is an aggressive malignancy with limited therapeutic options. The response to immune therapy is a determining factor for the prognosis of LUSC patients. This study aimed to develop a reliable immune-related prognostic signature in LUSC. We extracted gene expression and clinical data of LUSC from The Cancer Genome Atlas (TCGA). A total of 502 patients enrolled and were divided into respond and non-responder groups by the TIDE algorithm. The CIBERSORT algorithm and the LM22 gene signature were used to analyze the distribution of immune cells in LUSC. Efficacy and response strength of immunotherapy are calculated by the tumor mutation burden (TMB) and ESTIMATE Score. Differentially expressed genes (DEGs) between the two groups were analyzed. The differential expression genes related to overall survival were pointed as hub DEGs, and a prognostic signature was constructed with lasso regression analysis. LUSC patients were divided into responder and non-responder groups based on the response to immunotherapy. The distribution of immune cells was significantly different between the two groups. Forty-four DGEs were considered as overall survival-related genes. A prognostic signature was constructed, consisting of 11 hub-DGEs, including MMP20, C18orf26, CASP14, FAM71E2, OPN4, CGB5, DIRC1, C9orf11, SPATA8, C9orf144B, and ZCCHC5. The signature can accurately distinguish LUSC patients into high and low-risk groups. Moreover, the high-risk group had a shorter survival time than the low-risk group. The area under the ROC curve was 0.67. The multivariate Cox regression showed that the risk score calculated by the constructed signature was an independent prognostic predictor for LUSC patients. In short, we established a novel immune-related prognostic signature in LUCS, which has significant sensitivity and accuracy in predicting the prognosis of patients. Our research can guide the evaluation of the prognosis of LUSC patients in clinical, and the discovered immune-related genes can provide a theoretical basis for the discovery of new therapeutic targets.

## Introduction

Worldwide, lung cancer remains the deadliest malignancy which includes lung adenocarcinoma cancer (LUAD), lung squamous cell carcinoma (LUSC), large cell carcinoma (LCC), and small cell lung cancer (SCLC). LUSC is the second-largest subtype of non-small cell lung cancer. Due to the lack of distinct driver mutation and poor response to target therapy, the overall survival of LUSC patients is about 30% shorter than other NSCLC subtypes^1^.

More recently, immunotherapy has been applied in the treatment of lung cancer. Immune checkpoint inhibitors (ICIs) such as anti-PD-1/PDL-1 showed distinct efficacy (~ 30% response rate) and improved the overall survival of patients with metastatic NSCLC^2^. While, studies reported that tumor could monitor its surrounding environment to facilitate its proliferation, invasion, and metastasis^3^. The tumor microenvironment (TME), which including stromal cells, fibroblasts, endothelial cells, innate immune cells, and adaptive immune cells, has been reported to affect the prognosis of cancer patients. For instance, less T CD8^+^ infiltration was associated with a higher risk of brain metastases in NSCLC patients^4^. Tumor-associated macrophages (TAMs) are a well-known primary element of the tumor microenvironment and are also characterized as M2-like macrophages. Hwang et al. found that the elevated M2 ratio (CD163^+^/CD68^+^) was significantly related to poor overall survival in NSCLC, providing insight into TAM-based immunotherapy strategies^5^. Hence, systematic analyses of TME provide a novel strategy for tumor immunotherapy.

In the era of information explosion, bioinformatics can integrate multi-omics data, discover valuable features, and open up broader fields based on previous basic research. Currently, various bioinformatics algorithms are developed to predict TME and immunotherapy response, such as CIBERSORT, Timer, and ESTIMATE^6–8^. By analyzing information from large public datasets such as TCGA and GEO, we were able to explore potential mechanisms affecting the tumor microenvironment and immune responses in lung cancer patients. For example, Sun et al. developed an immune-related four-gene signature including ARNTL2, ECT2, PPIA, and TUBA4A, an independent prognostic factor for lung adenocarcinoma cancer (LUAD)^9^. Furthermore, Liu et al. built a prognostic model for NSCLC patients based on the expression profiles of autophagy-associated genes^10^. Therefore, it is indispensable to establish immune-related gene signatures and upgrade treatment strategies to improve patient survival.

In the current study, an 11 differential expressed hub genes (DEGs) prognostic signature was constructed, including MMP20, C18orf26, CASP14, FAM71E2, OPN4, CGB5, DIRC1, C9orf11, SPATA8, C9orf144B, and ZCCHC5. The signature showed moderate accuracy, and the risk score estimated by the signature is an independent prognostic indicator for LUSC.

## Materials and methods

### Data acquisition and TIDE analysis

RNA-sequencing and corresponding clinical data of LUSC were extracted from the TCGA database (https://portal.gdc.cancer.gov/)^11^. A total of 502 LUSC patients were enrolled in this study. Next, the Tumor Immune Dysfunction and Exclusion (TIDE) (http://tide.dfci.harvard.edu/) was used to predict the response to immunotherapy based on the simulation of tumor immune escape mechanism^12^. The response of the TCGA-LUSC cohort to immunotherapy based on TIDE algorithm divided patients with LUSC into non-responder and responder groups.

### The distribution of immune cells in LUSC based on the CIBERSORT method

The abundance of 22 immune cell types in LUSC was calculated by the CIBERSORT algorithm (https://cibersort.stanford.edu/)^6^, and the LM22 gene signature was used. The distribution of immune cells was analyzed in LUSC patients between non-responder and responder groups with unpaired Student t-tests, including B cells naïve, B cells memory, plasma cells, T cells CD8, T cells CD4 naïve, T cells CD4 memory resting, T cells CD4 memory activated, T cells follicular helper, T cells regulatory (Tregs), T cells gamma delta, NK cells resting, NK cells activated, monocytes, macrophages M0, macrophages M1, macrophages M2, dendritic cells resting, dendritic cells activated, mast cells resting, mast cells activated, eosinophils, and neutrophils.

### TMB and Tumor purity analysis

Tumor mutation burden (TMB) was identified as the total somatic nonsynonymous mutation counts in coding regions. TMB score of LUSC patients between responder and non-responder was calculated by Maftools R package with unpaired Student t-tests. The ESTIMATE algorithm (https://bioinformatics.mdanderson.org/estimate/) was applied to predict tumor immune infiltration levels using gene expression data^8^. From the algorithm, three scores will obtain Immune Score (the infiltration of immune cells in tumor tissue), Stromal Score (the presence of stroma in tumor tissue), and ESTIMATE Score (the tumor purity). A higher immune score represents higher infiltration status. The ESTIMATE Score in LUSC patients between responder and non-responder was calculated by Estimate R package (version 3.5.1) with unpaired Student t-tests.

### Identification DEGs between responder and non-responder

The limma package (https://www.bioconductor.org/packages/release/bioc/html/limma.html) was used to identify the DEGs in LUSC between responder and non-responder^13^. *P*-value < 0.05, false discovery rate (FDR) filter <  = 0.05, and Log (fold change) filter >  = 0.58 were considerate as the selected criteria of DEGs.

### Functional enrichment and pathway analysis

Gene Ontology (GO) and Kyoto Encylopedia of Genes and Genomes (KEGG) analysis Clusterprofiler software performed GO function enrichment analysis and KEGG pathway enrichment analysis in DEGs^14–16^. The significantly enriched pathways of those DEGs were clustered with CooLGeN (http://ci.smu.edu.cn/CooLGeN/Home.php).

Cytoscape ClueGo was used to explore the biological processes (BPs) and cellular components (CCs) enriched in selected DEGs (two‐sided hypergeometric test, adjusted *p* < 0 0.05 corrected with Benjamini–Hochberg).

### Related transcription factor exploring

TFs or sequence-specific DNA-binding factors were a cluster of proteins that could control the rate of transcription from DNA to mRNA, which can be obtained from the Cistrome Cancer database (http://cistrome.org/db/)^17^. TF gene expressions from the TCGA database were matched with the Cistrome Cancer database. Cytoscape software visually presented the TF-DEGs network based on the standard of correlation coefficient filter > 0.4 and *p*-value filter < 0.05. Overall survival analysis of those DEGs in LUSC was constructed by Kaplan–Meier plotter (http://kmplot.com/private/index.php.p=home).

### Lasso regression construction and verification for LUSC

Further, the DEGs were selected to construct a prognosis-related signature. The lung cancer samples were divided into two groups according to the median value of risk score (high-risk score group and low-risk score group). The Kaplan–Meier method was used to evaluate the availability of a prognostic model between the high-risk score group and the low-risk score group. Principal component analysis (PCA) and receiver operating characteristic (ROC) curves were used to test the classification measurement based on the risk score^18^. The distribution of immune cells was analyzed in LUSC patients between high and low-risk score groups. The clinical data were obtained from the TCGA database, including gender (male and female), age (aged <  = 65 and > 65), anatomic subdivision (L-lower, L- middle, L-upper, R-lower, R-middle, and R-upper), follow-up outcome (partial remission/response, complete remission/response, progressive disease, and stable disease), number pack-years smoked (packs from 0.15 to 240), pathologic T (tumor size, including T1, T2, T3, T4, and TX ), pathologic M (tumor metastasis, including M0, M1, and MX), pathologic N (tumor lymph node metastasis, including N0, N1, N2, and NX), pathologic stage (Stages I, II, III, and IV), person neoplasm cancer status (tumor or tumor-free), radiation therapy (no or yes), targeted molecular therapy (no or yes), and status (alive or dead). Clinic correlation between high-risk score group and low-risk score group was performed using heatmap R package. In addition, clinical characteristics (including age at initial diagnosis, anatomic subdivision, follow-up, gender, number pack-years smoked, pathologic M, pathologic N, pathologic T, pathologic stage, cancer status, radiation therapy, targeted molecular therapy) associated with overall survival were analyzed in lung cancer patients with univariate and multivariate Cox regression model.

### Statistical analysis

All the statistical analyses were performed using the R package (Vision3.5.1). For between-group comparisons, for normally distributed variables, the *p*-value was calculated with unpaired Student t-tests; and for non-normally distributed variables, the *p*-value was calculated with Mann–Whitney U tests (namely, the Wilcoxon rank-sum test), and statistical significance was set as *p* < 0.05. FDR and Benjamini–Hochberg for multiple testing were used for correction of the *p*-value in DEGs, GO, and pathway analyses. The Kaplan–Meier method was for the generation of survival curves. The Log-rank (Mantel-Cox) test was used to evaluate the statistical significance of differences, with a statistical significance of *p* < 0.05. The hazard ratio was calculated for univariate or and multivariate Cox proportional hazard regression models (*p* < 0.05).

## Results

### Responder and non-responder in LUSC

**Figure 1:**
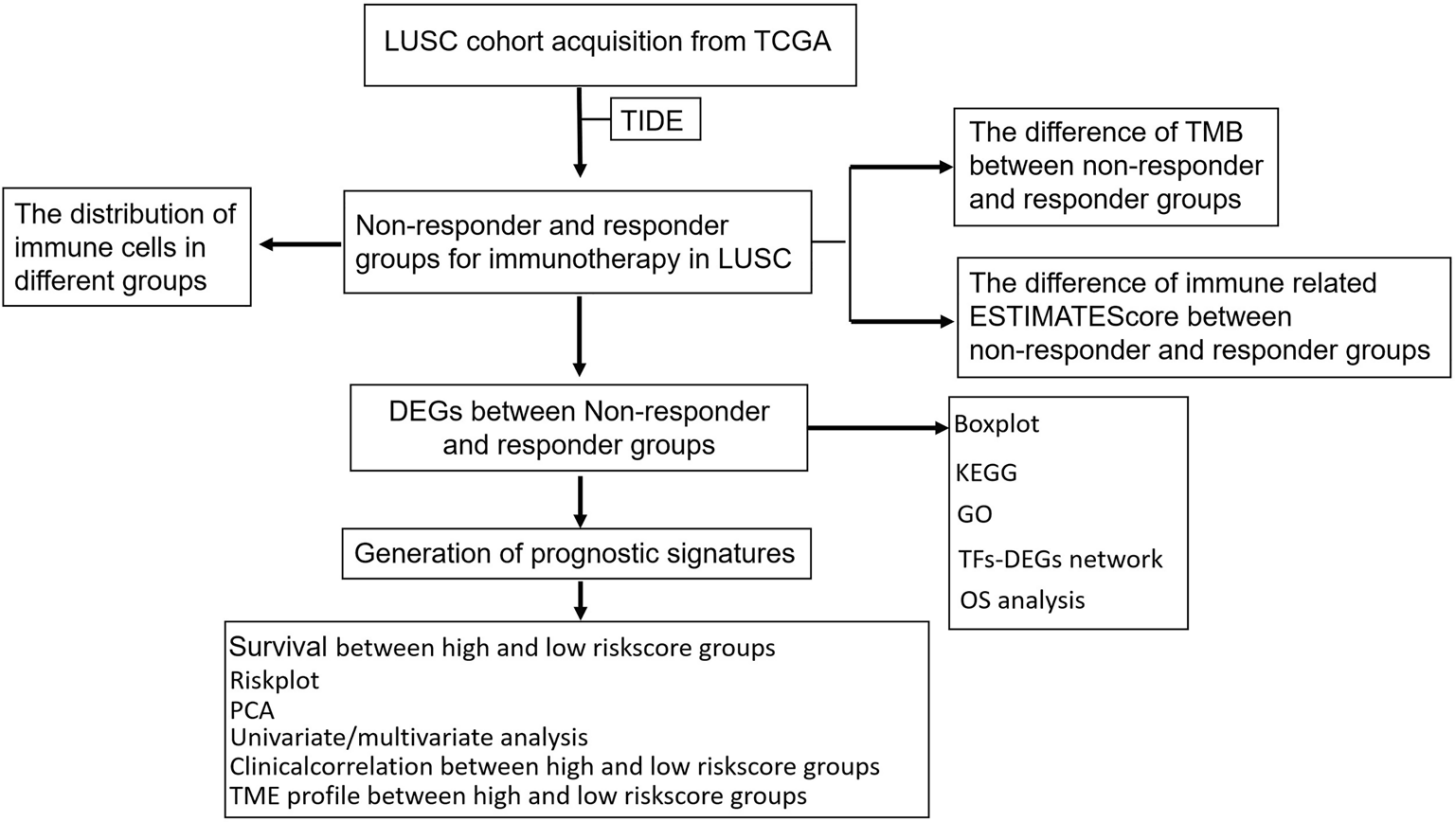
Flow chart for the construct and validation of a DEGs related prognosis signature.

**Figure 2:**
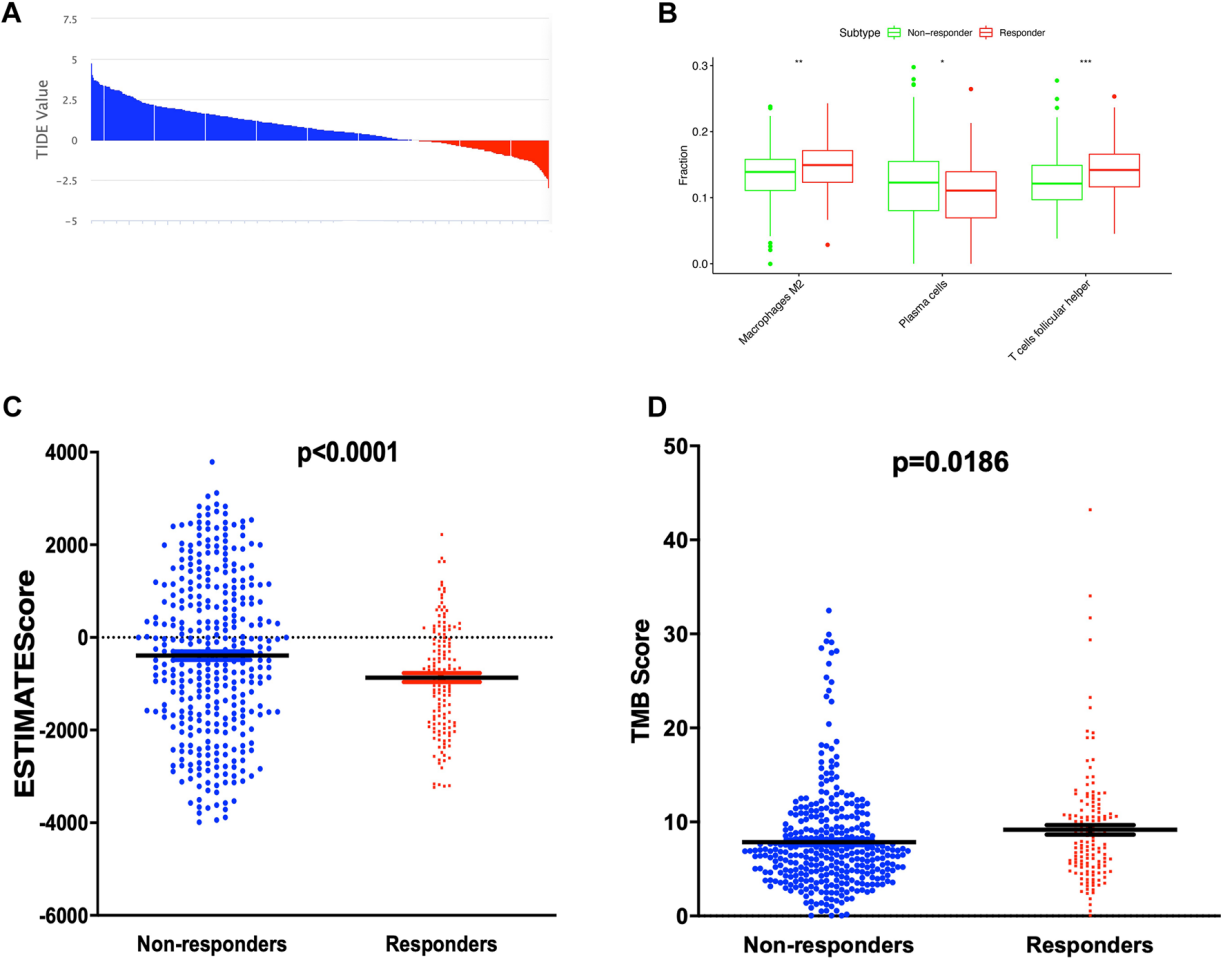
The immune-related characteristics between responder and non-responder. (**A**) 502 LUSC patients were divided into responder (red) and non-responder (blue) groups by the TIDE algorithm. (**B**) Boxplot showed the ratio differentiation of 3 kinds of immune cells between responder and non-responder groups. (**C**) Different ESTIMATE Score between responder and non-responder groups. (**D**) Different TMB score between responder and non-responder groups. *p < 0.05, **p < 0.01, and ***p < 0.001.

We conducted the study as described in the flow chart (Fig. 1). The RNA-sequencing data of 502 LUSC patients were extracted from TCGA (Supplementary Tables 1A, 1B, and 1C). In the TIDE algorithm prediction of immunotherapy response, the 502 LUSC patients were divided into responder (*n* = 147) and non-responder (*n* = 355) groups. (Fig. 2A, Supplementary Tables 2). We then used CIBERSORT to calculate the abundance of cell infiltration in the tumor microenvironment for each LUSC RNA expression data (Supplementary Table 3), such as B cells naïve, B cells memory, plasma cells, T cells CD8, T cells CD4 memory resting, T cells CD4 memory activated et al. The distribution of macrophages M2 and T cells follicular helper was significantly higher in responder group, while the distribution of plasma cell showed a decreased trend in responder group compared to non-responder, indicating that the patients in the response group may mainly rely on cellular immunity to kill tumors (Fig. 2B). The ESTIMATE R package calculated the ESTIMATE scores of the two groups, and the results showed that the ESTIMATE scores of the non-responding group were higher than the responding group (*p* < 0.0001, Fig. 2C), indicating higher tumor purity and distribution of infiltrating stromal and immune cells in the responding group. Furthermore, the responder group showed a significantly higher TMB score (*p* = 0.0186, Fig. 2D, Supplementary Tables 4).

### The identification of DEGs

**Figure 3:**
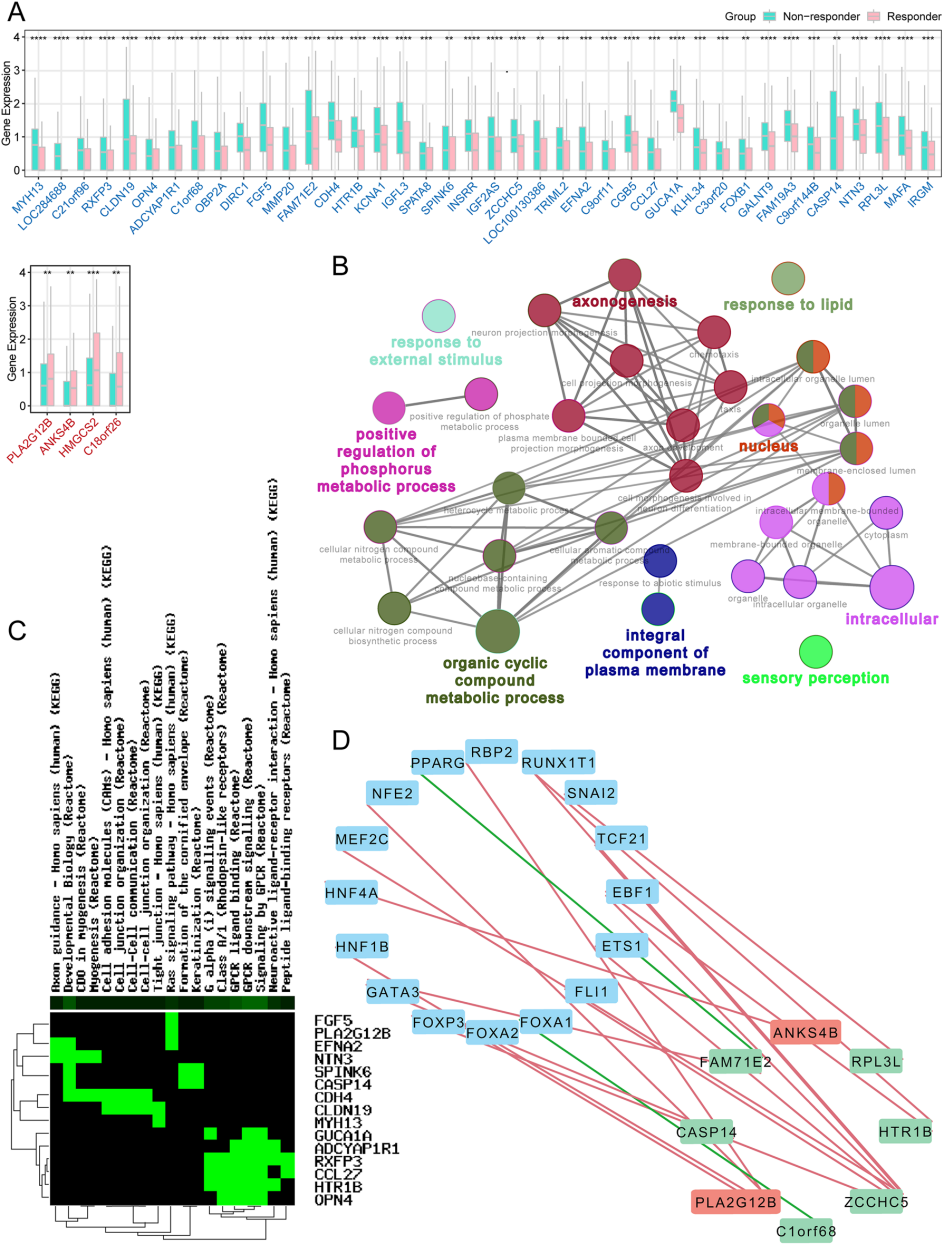
GO and KEGG enrichment analysis (**A**) Boxplot showed the 44 DEGs between responder and non-responder groups. (**B**) Go analysis of the 44 DEGs. (**C**) KEGG pathway analysis of the DEGs. (**D**) Regulatory network of TFs-DEGs. The DEGs in red showed high expression, and green showed low expression. The red line showed a positive correlation, and the green line showed a negative correlation.

To explore the potential biological feature under the responder group and non-responder group, a total of 44 DEGs were obtained between the two subtypes in LUSC (Supplementary Table 5). Four upregulated DEGs (including PLA2G12B, ANKS4B, HMGCS2, and C18orf26) and forty downregulated DEGs (including MYH13, LOC284688, C21orf96, RXFP3, CLDN19, OPN4, ADCYAP1R1, C1orf68, OBP2A, DIRC1, FGF5, MMP20, FAM71E2, CDH4, HTR1B, KCNA1, IGFL3, SPATA8, SPINK6, INSRR, IGF2AS, ZCCHC5, LOC100130386, TRIML2, EFNA2, C9orf11, CGB5, CCL27, GUCA1A, KLHL34, C3orf20, FOXB1, GALNT9, FAM19A3, C9orf144B, CASP14, NTN3, RPL3L, MAFA, and IRGM) were revealed (Fig. 3A).

### Enrichment analysis of DEGs

Then GO, and KEGG enrichment analyses were performed on these DEGs. We got 20 enrichments according to BP and 18 enrichments according to CC (Supplementary Table 6). The less *p* -value and more significant enrichment were shown with the greater node size. The same color indicated the same function group. As shown in Fig. 3B, nucleus DEGs were mainly enriched in response to lipid (ADCYAP1R1, DYNAP, HTR1B, IRGM, and TRIML2), axonogenesis (CDH4, EFNA2, FOXB1, and NTN3), positive regulation of phosphorus metabolic process (ADCYAP1R1, CLDN19, DYNAP, GUCA1A, INSRR, IRGM), organic cyclic compound metabolic process (FOXB1, GUCA1A, HMGCS2, and MAFA), sensory perception (CLDN19, GUCA1A, KCNA1, OBP2A, and OPN4), nucleus (CASP14, CLDN19, FAM205A, FOXB1, MAFA, RTL3, and TRIML2), integral component of the plasma membrane (ADCYAP1R1, CDH4, HTR1B, INSRR, KCNA1, OPN4, and RXFP3), intracellular (ADCYAP1R1, C3orf20, CASP14, CLDN19, DYNAP, EQTN, FAM205A, FOXB1, GALNT9, HMGCS2, HTR1B, IRGM, KCNA1, MAFA, MYH13, NTN3, RPL3L, RTL3, and TRIML2). KEGG enrichment analysis obtained 19 typical pathways, including CDO in myogenesis, myogenesis, GPCR ligand binding, Class A/1 (Rhodopsin-like receptors), developmental biology, G alpha (i) signaling events, cell–cell junction organization, Ras signaling pathway, formation of the cornified envelope, cell junction organization, neuroactive ligand-receptor interaction, GPCR downstream signaling, signaling by GPCR, Keratinization, cell–cell communication, cell adhesion molecules (CAMs), axon guidance, tight junction, peptide ligand-binding receptors (Fig. 3C and Supplementary Table 7). ADCYAP1R1 is a member of the GPCRs and has been shown to play a key role in nervous system. The activation of ADCYAP1R1 lead some downstream signal transduction pathways, such as MEK/ERK and Akt pathway, and delay apoptotic events thus enhancing cell survival^19^. Ras signaling pathway is recognized as one of the crucial pathways in tumorigenesis. Mutated ras genes stimulate cell proliferation and inhibit apoptosis of tumor cells. Targeted therapies based on RAS-mediated signaling inhibition have also attracted great attention. Studies have shown that sotorasib (KRAS G12C inhibitors) bring effective antitumor activity in lung cancer patients with KRAS G12C mutation^20^. Moreover, researchers suggested that the PD-L1 upregulation may be one of the main mechanisms of immune escape in KRAS-mutated NSCLC^21^. The combination of MAPK/ERK inhibitors and PD-1/PD-L1 inhibitors may be a novel strategy to overcome the EGFR-TKIs resistance with high PD-L1 expression. Further investigations of the DEGs and related pathways might contribute to therapeutic of LUSC patients.

### Regulatory network of transcription factors for DEGs

A total of 318 TF gene expressions from the TCGA database were matched with the Cistrome Cancer database (Supplementary Table 8). The associations between TFs and DEGs were based on the correlation coefficient filter > 0.4 and *p*-value filter < 0.05. After the final screening, 19 pairs of TFs-DEGs were identified based on co-expression analysis (Supplementary Table 9and Fig. 3D). Including 17 positive correlation coefficients (EBF1 and HTR1B, EBF1 and ZCCHC5, ETS1 and ZCCHC5, FLI1 and ZCCHC5, FOXA2 and PLA2G12B, FOXP3 and ZCCHC5, GATA3 and CASP14, GATA3 and FAM71E2, HNF1B and PLA2G12B, HNF4A and ANKS4B, MEF2C and ZCCHC5, NFE2 and PLA2G12B, RBP2 and PLA2G12B, RUNX1T1 and HTR1B, RUNX1T1 and ZCCHC5, SNAI2 and RPL3L, TCF21 and ZCCHC5) and two negative correlation coefficients (FOXA1 and C1orf68, PPARG and FAM71E2).

### Overall survival analysis of DEGs between responder and non-responder groups in LUSC

**Figure 4:**
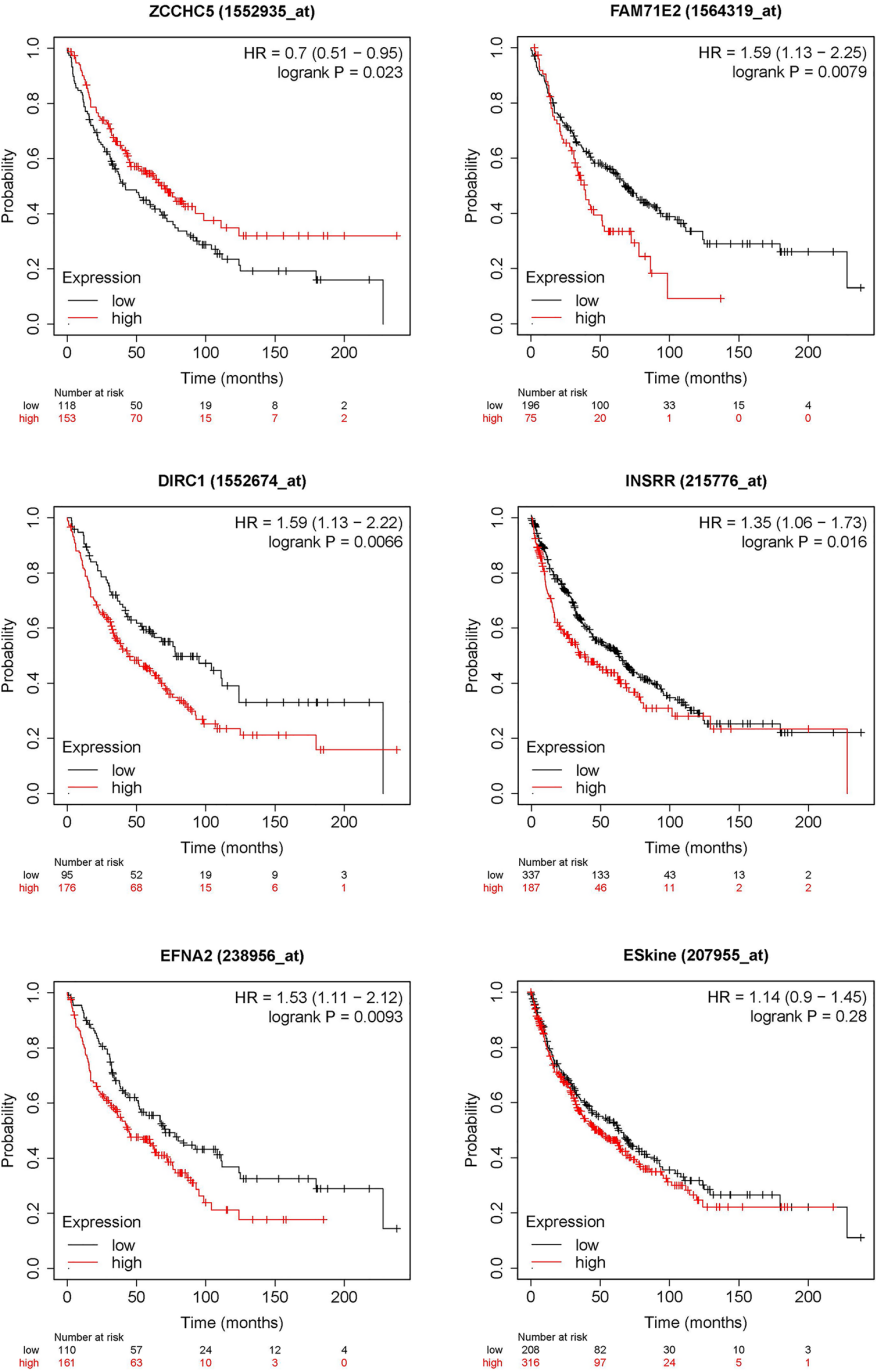

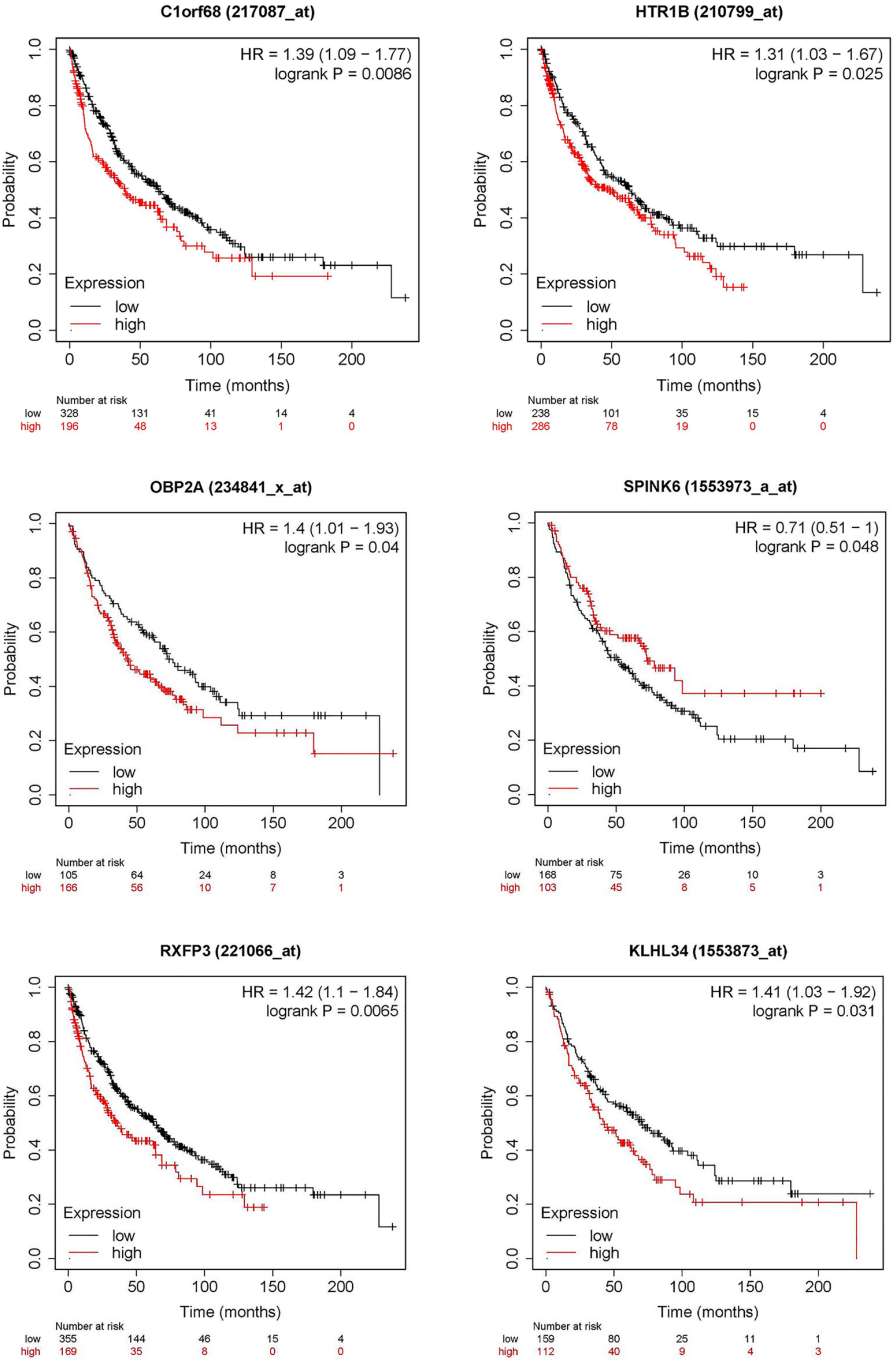

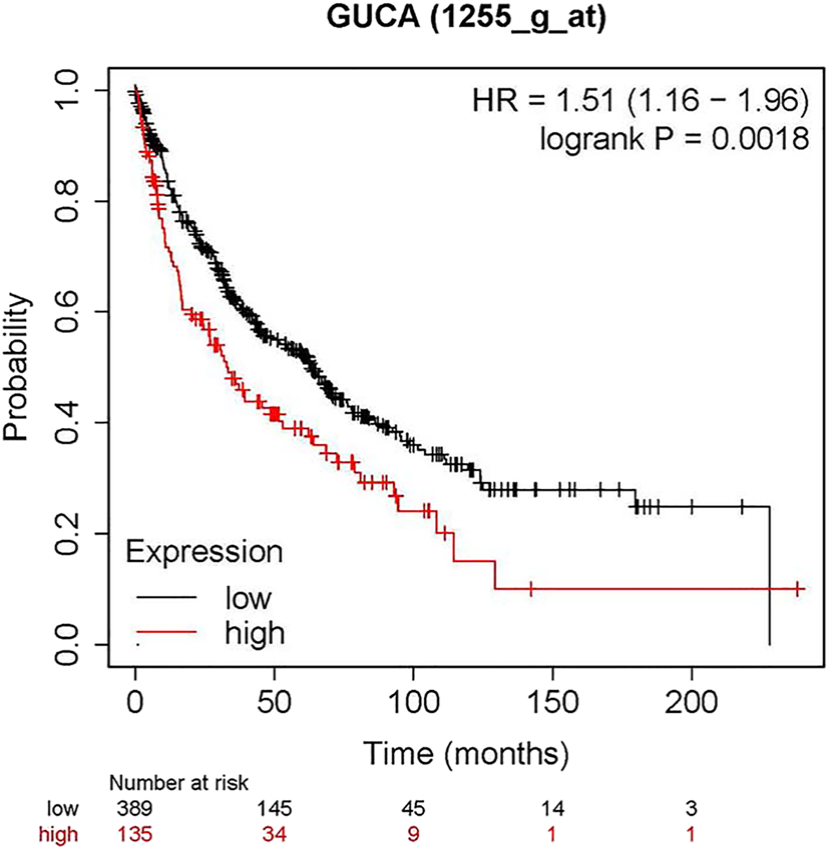
The analysis of overall survival-related DEGs.

The Kaplan–Meier plot analysis was performed to clarify the relation between the DEGs and LUSC overall survival. Among the 44 immune-related DEGs, 13 were significantly associated with overall survival of LUSC (*p* < 0.05). including ZCCHC5 (HR = 0.7, *p* = 0.023), FAM71E2 (HR = 1.59, *p* = 0.0079), DIRC1 (HR = 1.59, *p* = 0.0066), INSSR (HR = 1.35, *p* = 0.016), C1orf68 (HR = 1.39, *p* = 0.0086), EFNA2 (HR = 1.53, *p* = 0.0093), ESkine (HR = 1.14, *p* = 0.28), GUCA (HR = 1.51, *p* = 0.0018), HTR1B (HR = 1.31, *p* = 0.025), KLHL34 (HR = 1.41, *p* = 0.031), OBP2A (HR = 1.4, *p* = 0.04), RXFP3 (HR = 1.42, *p* = 0.0065), and SPINK6 (HR = 0.71, *p* = 0.048) in LUSC (Fig. 4). In the above results, FAM71E2, DIRC1, INSRR, EFNA2, ESkine, GUCA, HTR1B, KLHL34, OBP2A, RXFP3, and SPINK6 were under expressed in the responder group. It was very consistent that the LUSC patients with these genes downregulated showed a better prognosis because of a higher response to immunotherapy.

### Construction of prognostic model for LUSC based on lasso analysis

**Figure 5:**
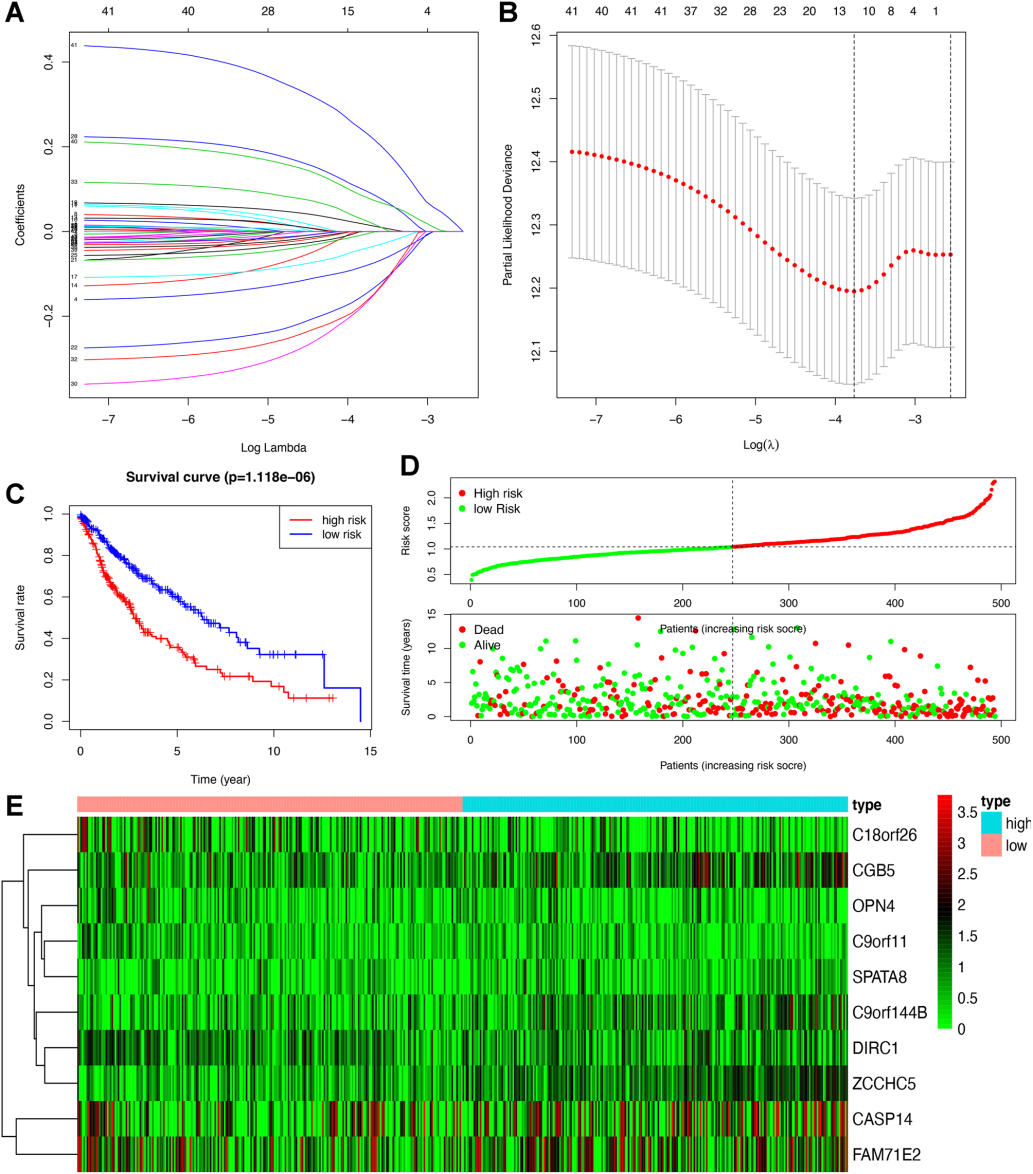
Constructed of 11 DGEs related prognosis signature. (**A** and **B**). Hub DEGs were selected by LASSO regression analysis. (**C**) Overall survival analysis between high-risk and low-risk score groups. (**D**) The individual inflection points of the risk score curve and risk score plot between high-risk and low-risk score groups. (**E**) Risk score heatmap of 11 hub DEGs.

We obtained the DEGs between the responder and non-responder groups and discovered their relation with LUSC overall survival from the above analysis. After the selection of lasso regression^22^, when log (lambda) was between − 3 and − 4, 11 out of 44 DEGs were defined as an ideal element of the immune-related DEGs signature model, including MMP20, C18orf26, CASP14, FAM71E2, OPN4, CGB5, DIRC1, C9orf11, SPATA8, C9orf144B, and ZCCHC5(Fig. 5A and B). The risk score for predicting prognostic risk in LUSC patients was then calculated with the following formula: Risk score = (− 0.08 × Exp MMP20) + (− 0.039 × Exp C18orf26) + (0.016 × Exp CASP14) + (− 0.005 × Exp FAM71E2) + (− 0.145 × Exp OPN4) + (0.0952 × Exp CGB5) + (− 0.167 × Exp DIRC1) + (− 0.163 × Exp C9orf11) + (0.0249 × Exp SPATA8) + (0.0805 × Exp C9orf144B) + (0.231 × Exp ZCCHC5). Subsequently, the LUSC patients were divided into high (*n* = 247) and low-risk score (*n* = 247) groups according to the mean value of risk scores (Supplementary Table 10). Figure 5C and D revealed that the low-risk score group obtained a significantly more favorable overall survival than the high-risk score group (Fig. 5C and D). The heatmap showed the different expressions of the identified genes in the prognosis model between high and low-risk score groups (Fig. 5E).

**Figure 6:**
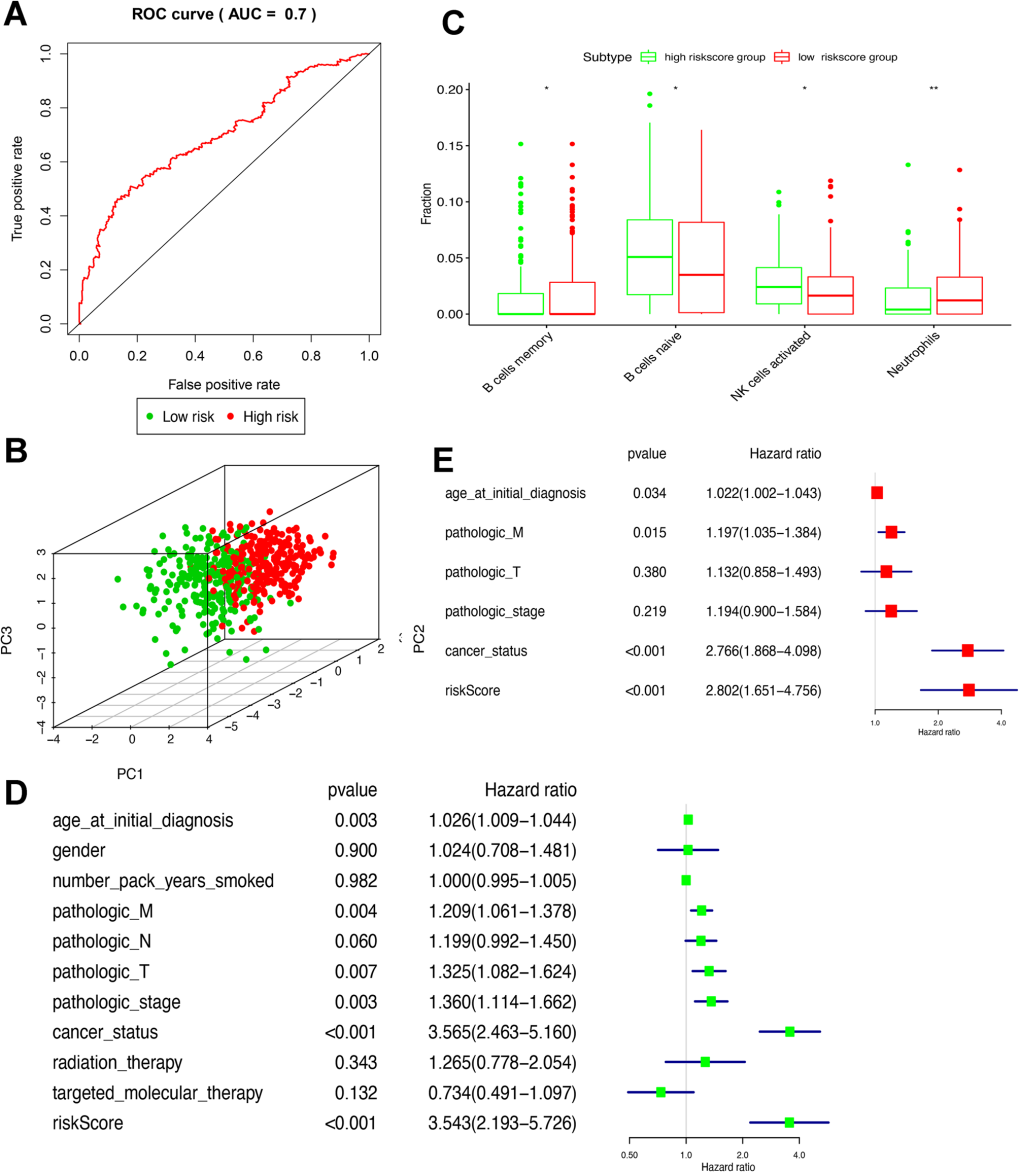
Validation of the signature (**A**) ROC curve analysis of the prognosis signature in TCGA dataset. (**B**) Principal component analysis. (**C**) Boxplot showed the ratio differentiation of 4 kinds of immune cells between high-risk score and low-risk score groups. DE Univariate and multivariate Cox regression analysis in LUSC. *p < 0.05, **p < 0.01.

Furthermore, the prediction value of this signature model was evaluated by PCA and ROC. The results showed that the AUC is 0.7 and all LUSC samples can be well divided into high-risk and low-risk groups (Fig. 6A and B). In addition, we assessed the distribution of immune cells between high and low-risk score groups in LUSC, B cells memory, B cells naïve, neutrophils, and NK cells activated were significantly differentially expressed. (Fig. 6C).

### Validation of the prognostic signature

**Figure 7:**
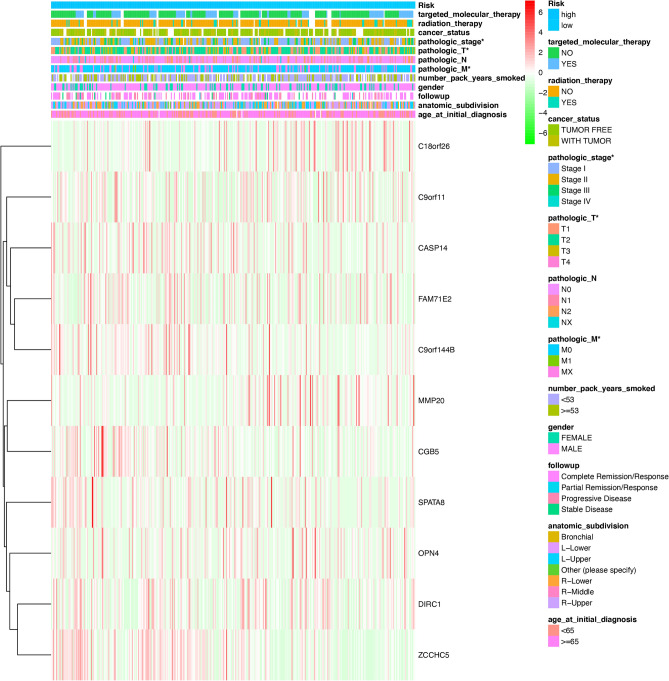
The heatmap of clinical characteristics between high-risk score and low-risk score groups. *p < 0.05.

The clinical data were obtained from the TCGA database (Supplementary Table 11), including gender (male and female), age (aged <  = 65 and > 65), anatomic subdivision (L-lower, L- middle, L-upper, R-lower, R-middle, and R-upper), follow-up outcome (partial remission/response, complete remission/response, progressive disease, and stable disease), number pack-years smoked (packs from 0.15 to 240), pathologic T (tumor size, including T1, T2, T3, T4, and TX ), pathologic M (tumor metastasis, including M0, M1, and MX), pathologic N (tumor lymph node metastasis, including N0, N1, N2, and NX), pathologic stage (Stages I, II, III, and IV), person neoplasm cancer status (tumor or tumor-free), radiation therapy (no or yes), targeted molecular therapy (no or yes), and status (alive or dead). The univariate Cox regression analysis revealed that age_at_initial_diagnosis, pathologic_M, pathologic_T, pathologic_stage, cancer status, and risk score were correlated significantly with overall survival (Fig. 6D). The multivariate Cox regression analysis revealed that age_at_initial_diagnosis, pathologic_M, cancer status, and risk score possibly acted as an independent risk factor in LUSC (Fig. 6E). The heatmap showed risk group had a significant association with clinical features, including pathologic T, pathologic M, and pathologic stage (Fig. 7).

## Discussion

Immunotherapy is identified as an effective therapeutic method in multiple cancers. Conventional agents such as anti-PD-L1/PD-1, anti-CTLA4, and anti-IL-2 showed good efficacy. However, due to tumor heterogeneity, microenvironment, and other factors, some patients experienced limited remission or even disease progression. Studies have shown that extrinsic factors such as tumor genetics, age, microbiota, and the presence of infectious agents et al. are necessary elements in response to immunotherapy^23^. Recently, with the development of the next-generation sequencing technology, the expression profile of cancer-related genes gradually enriched. In order to get out of the dilemma of tumor treatment and improve the efficacy and prognosis of tumor patients, increasing prognostic models have been constructed.

In this study, we identified a prognosis model that consisted of MMP20, C18orf26, CASP14, FAM71E2, OPN4, CGB5, DIRC1, C9orf11, SPATA8, C9orf144B, and ZCCHC5 between immune responder and non-responder groups. Aseervatham et al. reported that Matrix Metalloproteinase 20 (MMP20) played a tumorigenic role on Oral Squamous Cell Carcinomas (OSCCs) by upregulating the genes related to invasion, metastasis, angiogenesis, and epithelial-mesenchymal transition (EMT)^24^. They also elucidated that MMP20 and its cognate DSPP paring a potential marker of some epithelial cancers^25^. The expression of C18orf26 induced the activation of Akt and sustaining the high proliferation of tumor cells, making it a potential novel therapeutic target^26^. Handa et al. reported that the high CASP14 expression is associated with proliferation, cancer stemness of breast cancer^27^. Melanopsin (OPN4) was found to be involved in pigmentation, cell death induction, and molecular clock modulation, thereby regulating the cellular response to UVA radiation which is the critical cause of melanoma^28^. Moreover, Chen et al. discovered the relationship between a woman's placental homologous CGB5 and her post-pregnancy breast cancer risk, with women carrying the variant C allele of CGB5 rs726002 earlier age at childbirth suffer a higher breast cancer risk^29^. Disrupted in Renal Cancer 1 (DIRC1) was related to tumor progression and poor prognosis in gastric cancer^30^. Chorionic gonadotrophin beta5 (CBG5) was also identified as a DEG in prognosis signatures for gastric cancer^31,32^. Dysregulation of RNA-binding proteins was associated with tumorigenesis. A previous study reported that ZCCHC5 is a prognosis-associated hub gene in LUSC, which is consistent with our findings^33^. Although some of these 11 genes have not been reported to be directly related to tumor development, our study has identified their potential as new tumor therapeutic targets.

In terms of immune-related pathways, DEGs between responder and the non-responder group were mainly enriched in including CDO in myogenesis, myogenesis, GPCR ligand binding, Class A/1 (Rhodopsin-like receptors), developmental biology, G alpha (i) signaling events, cell–cell junction organization, Ras signaling pathway, formation of the cornified envelope, cell junction organization, neuroactive ligand-receptor interaction, GPCR downstream signaling, signaling by GPCR, Keratinization, cell–cell communication, cell adhesion molecules (CAMs), axon guidance, tight junction, peptide ligand-binding receptors. G-protein–coupled receptors (GPCRs) is an extensive family of cell surface receptors in the human genome and play distinct roles in tumorigenesis. GPCRs are pleiotropic to the cell signal proteins they activate, and different ligands can induce specific receptor conformational states after activating. Several conformation changes in a single GPCR may produce discrete downstream signaling pathways^34^. Thus, the concept of multidrug combination shifts from one drug per GPCR target. The innate and adaptive immune responses depend on the dynamic control of leukocytes, and leukocytes receive a variety of molecular signals through GPCRs^35^. Recently, studies have shown that the effect on leukocyte migration is also thought to be the function in “Multiple GPCRs systems"^36^. Additionally, GPCR was functional as guidance for T cells to the target area^36^. From our enrichment analysis, the DEGs were significantly related to GPCRs (GPCR ligand binding, GPCR downstream signaling, signaling by GPCR). It suggested that GPCRs might be closely related to the immunotherapy response in LUSC patients. Furthermore, the Ras signaling pathway is also a frequent tumor-related signaling pathway. In summary, the immune-related gene signature is biologically significant in LUSC.

Dysregulation of transcription factors is a significant cause in malignant tumors, and therapeutic strategies targeting TFs have specific therapeutic effects on diseases related to immunotherapy^37^. Small molecule drugs could regulate the function of transcription factors by directly targeting their structurally and functionally domains^38^.It is of great significance to fully reveal the regulatory network of TFs-DEGs. Nineteen pairs of TFs-DEGs were identified based on co-expression analysis. The transcription factor Early B cell factor 1 (EBF1) is expressed in early B cells and recognized as an upstream transcription factor of potential oncogene PNO1^39,40^. EBF1 inhibits cell proliferation and induces cell apoptosis in colorectal cancer (CRC) cells by inhibiting the activation of the PNO1-mediated p53/p21 signaling pathway^40^. GATA3 has conventionally been described as a T helper 2 cell differentiation driver and functions as an immune regulator^41^. Other identified transcription factors have also been reported to be associated with tumors, such as FOXA2 in oral cancer and endometrial cancers, HNF1B in prostate cancer^42–44^. In our results, EBF1 was positively corelated with HTR1B and ZCCHC5, GATA3 was positively related with CASP14 and FAM71E2, and FOXA2 was also positively related with ZCCHC5. These all suggested that identified DEGs and TFs could be latent biomarkers or therapeutic targets for LUSC.

Moreover, infiltrating inflammatory cells such as neutrophils were identified as an important regulator in the tumor microenvironment and associated with tumor initiation, proliferation, and metastasis^45^. Studies reported that neutrophils promote the antitumor immunity in CRC by enhancing the responsiveness of CD8 + T cells to TCR triggering^46^. Similarly, Eruslanov et al. pointed that the tumor-associated neutrophils (TANs) stimulate T cells responses in the earliest stages of lung cancer^47^. In this study, the higher proportion of neutrophils in the low-risk score group may also be related to the enhanced immune response of neutrophils.

In the diagnosis and treatment of cancer, increasing clinical data suggests that limited effect was obtained from the single biomarkers and target. The combination of multiple indicators and personalized treatment options leads to better outcomes for patients.

Most recently, there are studies on immune-related genes identify and signatures construction in LUSC. Gu et al. verified the prognostic-related and immune checkpoints-related genes in LUSC patients^48^. And other two studies are both built immune-related signatures of LUSC. Fan et al. finally validated the predictive value of the signature by tissue sample and that's what we need to do with the rest of our study^49^. Additionally, Hou and his colleagues also discussed the potential upstream regulator of DEGs—transcription factors, but lacked functional enrichment and pathway analysis^50^. These studies, including ours, may provide a theoretical basis for more effective, individualized immunotherapy.

The study needs to be improved. A large prospective clinical study is necessary to verify the signature. Next, in vitro and in vivo functional assays are needed to clarify the role of DEG further.

## Conclusion

In conclusion, the DEGs prognosis signature is biologically significant in LUSC. Moreover, the multivariate Cox regression showed that the risk score calculated by the 11 hub DEGs signature was an independent prognostic predictor for LUSC patients.

## Supplementary Information

Supplementary Information 1.

Supplementary Information 2.

Supplementary Information 3.

Supplementary Information 4.

Supplementary Information 5.

Supplementary Information 6.

Supplementary Information 7.

Supplementary Information 8.

Supplementary Information 9.

Supplementary Information 10.

Supplementary Information 11.

## Data Availability

The datasets analysed during the current study are available in the TCGA (https://portal.gdc.cancer.gov/) or available upon request by contact with the corresponding author.
